# Estimating correlation between multivariate longitudinal data in the presence of heterogeneity

**DOI:** 10.1186/s12874-017-0398-1

**Published:** 2017-08-17

**Authors:** Feng Gao, J. Philip Miller, Chengjie Xiong, Jingqin Luo, Julia A. Beiser, Ling Chen, Mae O. Gordon

**Affiliations:** 1 Department of Surgery, Division of Public Health Sciences, Washington University School of Medicine, 660 S. Euclid Ave., St. Louis, MO 63110 USA; 2 Division of Biostatistics, Washington University School of Medicine, 660 S. Euclid Ave., St. Louis, MO 63110 USA; 3 Department of Ophthalmology & Visual Sciences, Washington University School of Medicine, 660 S. Euclid Ave., St. Louis, MO 63110 USA

**Keywords:** Bivariate linear mixed model (BLMM), Multivariate longitudinal data, Correlation, Heterogeneity

## Abstract

**Background:**

Estimating correlation coefficients among outcomes is one of the most important analytical tasks in epidemiological and clinical research. Availability of multivariate longitudinal data presents a unique opportunity to assess joint evolution of outcomes over time. Bivariate linear mixed model (BLMM) provides a versatile tool with regard to assessing correlation. However, BLMMs often assume that all individuals are drawn from a single homogenous population where the individual trajectories are distributed smoothly around population average.

**Methods:**

Using longitudinal mean deviation (MD) and visual acuity (VA) from the Ocular Hypertension Treatment Study (OHTS), we demonstrated strategies to better understand the correlation between multivariate longitudinal data in the presence of potential heterogeneity. Conditional correlation (i.e., marginal correlation given random effects) was calculated to describe how the association between longitudinal outcomes evolved over time within specific subpopulation. The impact of heterogeneity on correlation was also assessed by simulated data.

**Results:**

There was a significant positive correlation in both random intercepts (ρ = 0.278, 95% CI: 0.121–0.420) and random slopes (ρ = 0.579, 95% CI: 0.349–0.810) between longitudinal MD and VA, and the strength of correlation constantly increased over time. However, conditional correlation and simulation studies revealed that the correlation was induced primarily by participants with rapid deteriorating MD who only accounted for a small fraction of total samples.

**Conclusion:**

Conditional correlation given random effects provides a robust estimate to describe the correlation between multivariate longitudinal data in the presence of unobserved heterogeneity (NCT00000125).

## Background

Estimating the correlation coefficients between outcome variables is one of the most important analytical tasks in epidemiological and clinical research. When independent observations are available for each outcome variable, Pearson’s correlation coefficients are often used. In many epidemiological and clinical studies, however, the same individuals are also followed repeatedly over time for a series of measurements with regarding to the collection of outcome variables. Such multivariate longitudinal data provide a unique opportunity to study the joint evolution of these outcomes over time. A simple Pearson’s correlation coefficient is no longer applicable for assessing correlation because a multivariate longitudinal model has to account for two types of correlations simultaneously, namely the serial correlation between observations at different time points within a subjects and the cross correlation between observations on different outcome variables at each time point [1].

During the last few decades, many statistical models have been proposed in statistical literature for the analysis of multivariate longitudinal data and the most popular one is the joint mixed model which links separate linear mixed models by allowing their model-specific random effects to be correlated [2]. The advantages of this approach include well-established theory [1], efficiency gains [3], and commercially available software packages for model fit [4, 5]. More importantly, a joint random-effect model allows assessing correlation between different outcomes. It can provide a succinct summary for not only how the evolution of one outcome variable is correlated to the evolution of another outcome (“association of evolution”), but also how the correlation between outcomes changes over time (“evolution of association”) [6].

Various extensions have been made in recent years in joint mixed models for a wide range of research fields [2, 7]. A typical joint mixed model often consists very few (usually 2 or 3) continuous outcome variables, takes a linear parametric functional form over time, holds a multivariate normality distribution for the vector of random effects, and assumes that the outcome variables are mutually independent given random effects. Fieuws and Verbeke [6] relaxed the conditional independence assumption by allowing the error components of the outcomes to be correlated. Gueorguieva and Sanacora [3] developed a joint mixed model to incorporate different types of longitudinal outcomes and analyzed repeated measurements on ordinal and continuous variables measuring the same underlying disease severity over time. Fieuws and Verbeke [8] took a pairwise fitting approach to construct the variance-covariance matrix for the joint distribution of random effects and assessed the hearing threshold profiles (22 outcomes) in a nature aging study. Putter et al. [9] proposed a latent class joint model (e.g., assuming the vector of random effects following a multinomial rather than normal distribution) to identify lung cancer patients with distinct patterns regarding their evolvement of denial over time and to assess the association between denial patterns and the trajectories of other physical and emotional longitudinal measurements. Recently Luo et al. [10] extended the model to non-longitudinal setting and proposed a bivariate linear mixed model to estimate correlation coefficients in cross-sectional data from a family-type clustered design. For other approaches in the analysis of multivariate longitudinal data as well as for more details in recent development, see the comprehensive reviews by McCulloch [7], Bandyopadhyay et al. [11], Verbeke et al. [2] and the references therein.

Almost all of the aforementioned joint mixed models (except Putter et al. [9]) also assume that subjects are drawn from a single homogenous population and that the individual trajectories are smoothly distributed around the population average. In this article, we intended to address the issue when such a one-size-fit-all assumption is violated, using participants with newly diagnosed primary open angle glaucoma (POAG) from the Ocular Hypertension Treatment Study (OHTS). POAG is a chronic progressive optic neuropathy and the rate of vision deterioration can vary substantially from patient to patient. For example, a natural history study on a cohort of patients newly diagnosed with glaucoma found out that the mean deviation (MD) index, a global summary measure for visual field test, in some patients can deteriorate at an alarming rate of 10 dB (dB) per year, while in others the MD virtually did not change in 6 years without any treatment [12]. It is therefore important to take potential heterogeneity into consideration when correlation is assessed.

In this article, we demonstrated strategies to assess the correlation between multivariate longitudinal data in the presence of potential heterogeneity. Specifically, bivariate linear mixed model (BLMM) was fitted to assess both the “association of evolution” (i.e., correlation between random effects) and “evolution of association” (i.e., marginal correlation over time), and then conditional correlation (i.e., marginal correlation given random effects) was calculated to describe how the association between longitudinal outcomes evolved over time within a “true” but unobserved subset of individuals. The disease heterogeneity was also approximated by subgroups (latent classes) from latent class analysis (LCA) where population variability is captured by differences across subgroups in the shape and level of their trajectories [13]. These subgroups were incorporated into BLMMs to further understand the impact of heterogeneity on correlation. Our method was similar to the latent class joint model by Putter et al. [9] except that our primary goal focused on correlations rather than trajectories. The remainder of this paper was structured as follows. Section 2 described the OHTS data in more detail. Section 3 specified the bivariate linear mixed model (BLMM) model to assess correlation among multivariate longitudinal data. The method was applied to data from OHTS in Section 4 and a simple simulation study was also performed to assess the impact of heterogeneity on correlation in Section 5. Finally, we concluded with a discussion in Section 6.

### Study cohort: Ocular hypertension treatment study (OHTS)

Between 1994 and 2009, the OHTS enrolled 1636 participants with ocular hypertension but with no evidence of glaucomatous damage and randomized to either observation or treatment with ocular hypotensive medication. The participants were followed for a median of 13 years and the disease progression was monitored regularly every 6 months. In OHTS, 362 eyes from 279 participants developed POAG during study and constituted the largest cohort of POAG with known date of diagnosis, with a median pre-diagnosis follow-up of 8 years and median post-diagnosis follow-up of 4.8 years. The design and methods of the OHTS have been described in detail elsewhere [14].

**Fig. 1:**
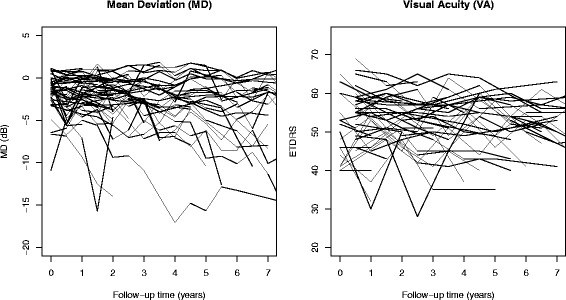
Individual trajectories of 50 randomly selected OHTS participants for mean deviation (MD) and visual acuity (VA), where time 0 represents the date of diagnosis

This paper only considered data measured during post-diagnosis period. As in many clinical trials on chronic diseases, multiple longitudinal outcomes were collected in OHTS to fully explore the multidimensional impairment caused by POAG. Our analysis restricted to 2 longitudinal outcomes, namely mean deviation (MD) and clinical visual acuity (VA). MD is a global index from Humphrey 30–2 visual field (VF) tests and reflects *functional damage*. VA is measured by the standard ETDRS acuity testing (ETDRS ranged 0 to 110, with the conventional 20/20 vision corresponding to ETDRS score of 100) and a low ETDRS score indicates a poor visual ability. MD was measured every 6 months while VA was measured annually. This study has been approved by Washington University Institutional Review Board. Our analysis cohort consisted the first eye of 269 OHTS participants who developed POAG and had at least 2 post-diagnosis measurements in both MD and VA. To reduce the potential influence of cases with greater attrition rates, we excluded all the measures taken 7.5 years after diagnosis and ended up with at least 30 observations in each outcome at any given time points. Figure 1 showed the raw data of 50 randomly selected participants and there was a considerable variability in the trajectories for both MD and VA. Besides these two longitudinal outcomes, following demographic and clinical characteristics were also included in the data: age at diagnosis (years), gender, race (African American vs. Others), randomization groups (Observation vs. Treatment), intraocular pressure (IOP, mmHg), central corneal thickness (CCT, μm), and horizontal cup/disc ratio (HCD).

## Methods

Since MD is arguably the most important index for monitoring POAG progression in clinical practice, in this article we focused on the heterogeneity of this pivotal variable and its impact on the correlation between MD and VA.

### Bivariate linear mixed model (BLMM) between MD and VA

Let Y_1i_(t) and Y_2i_(t) denote the MD and VA for i^th^ participant at time t, Z_i_ = {AGE, Gender, Race, Observation group, CCT, IOP, HCD} be the vector of baseline covariates of *i* ^th^ participant, then each outcome can be described by a linear mixed model,

$$ {\displaystyle \begin{array}{l}{\mathrm{Y}}_{1\mathrm{i}}(t)={Z}_i{\alpha}_Z+\left({\alpha}_0+{\alpha}_{0i}\right)+\left({\alpha}_1+{\alpha}_{1i}\right){t}_{ij}+{\varepsilon}_{1i}(t),\\ {}\ {\mathrm{Y}}_{2\mathrm{i}}(t)={Z}_i{\beta}_Z+\left({\beta}_0+{\beta}_{0i}\right)+\left({\beta}_1+{\beta}_{1i}\right){t}_{ij}+{\varepsilon}_{2i}(t).\end{array}} $$

Where {*α*_0_, *β*_0_} is the vector of intercepts, {*α*_1_, *β*_1_}is the vector of slopes during post-diagnosis period, while {*α*_*Z*_, *β*_*Z*_} is the vector of fixed effects for all other covariates at diagnosis. {*a*_0*i*_, *a*_1*i*_}and{*b*_0*i*_, *b*_1*i*_, *b*_2*i*_}represent random intercept and slope for longitudinal MD and VA respectively, and the BLMM is constructed by linking the two outcomes via a joint distribution of the random effects,

$$ \left[\begin{array}{c}\hfill {a}_{0i}\hfill \\ {}\hfill {b}_{0i}\hfill \\ {}\hfill {a}_{1i}\hfill \\ {}\hfill {b}_{1i}\hfill \end{array}\right]\kern0.5em \sim N\left(0,\varSigma \right),\kern0.75em \varSigma =\left[\begin{array}{cccc}\hfill {\sigma}_{a_0}^2\hfill & \hfill {\sigma}_{a_0{b}_0}\hfill & \hfill {\sigma}_{a_0{a}_1}\hfill & \hfill {\sigma}_{a_0{b}_1}\hfill \\ {}\hfill \hfill & \hfill {\sigma}_{b_0}^2\hfill & \hfill {\sigma}_{a_1{b}_0}\hfill & \hfill {\sigma}_{b_0{b}_1}\hfill \\ {}\hfill \hfill & \hfill \hfill & \hfill {\sigma}_{a_1}^2\hfill & \hfill {\sigma}_{a_1{b}_1}\hfill \\ {}\hfill \hfill & \hfill \hfill & \hfill \hfill & \hfill {\sigma}_{b_1}^2\hfill \end{array}\right] $$

The error terms assume following bivariate normal distribution and independent of random effects,

$$ \left[\begin{array}{c}\hfill {\varepsilon}_{1i}\hfill \\ {}\hfill {\varepsilon}_{2i}\hfill \end{array}\right]\sim N\left(\left[\begin{array}{c}\hfill 0\hfill \\ {}\hfill 0\hfill \end{array}\right],\left[\begin{array}{cc}\hfill {\sigma}_1^2\hfill & \hfill 0\hfill \\ {}\hfill 0\hfill & \hfill {\sigma}_2^2\hfill \end{array}\right]\right). $$

### Estimated correlations between longitudinal MD and VA

Once the BLMM has been fitted and the variance-covariance matrix is obtained, the correlation between MD and VA can be calculated analytically and following 3 different types of correlations are estimated in this paper.

#### Association of evolution (correlation between random effects)

The correlation between random effects (intercepts and post-diagnosis slopes) summarizes how the evolution of MD is associated with the evolution of VA [6],

$$ {\rho}_{\mathrm{intercept}}=\frac{\sigma_{a_0{b}_0}}{\sqrt{\sigma_{a_0}^2}\sqrt{\sigma_{b_0}^2}},\kern1em \mathrm{and}\kern0.75em {\rho}_{\mathrm{slope}}=\frac{\sigma_{a_1{b}_1}}{\sqrt{\sigma_{a_1}^2}\sqrt{\sigma_{b_1}^2}}\kern1.25em . $$

#### Evolution of association (marginal correlation)

The marginal correlation during post-diagnosis period allows answering the question how the association between MD and VA evolves over time [6],

$$ {\rho}_{\mathrm{marginal}}(t)=\frac{\sigma_{a_0{b}_0}+t{\sigma}_{a_0{b}_1}+t{\sigma}_{a_1{b}_0}+{t}^2{\sigma}_{a_1{b}_1}}{\sqrt{\sigma_{a_0}^2+2t{\sigma}_{a_0{a}_1}+{t}^2{\sigma}_{a_1}^2+{\sigma}_1^2}\sqrt{\sigma_{b_0}^2+2t{\sigma}_{b_0{b}_1}+{t}^2{\sigma}_{b_1}^2+{\sigma}_2^2}}. $$

#### Conditional correlation (marginal correlation given random effects)

The conditional marginal correlation given specific random effects allows us to assess how the association between MD and VA evolves over time within certain clinically relevant subgroups. Since a majority of POAG participants tends to have a relatively stable vision function for a long period over time after initial diagnosis, the correlation between MD and VA in the participants with stable vision would provide a useful tool to assess the impact of heterogeneity. Let *γ*_0*i*_ = *Z*_*i*_ *α*_*Z*_ + *α*_0_ + *a*_0*i*_ and *γ*_1*i*_ = *α*_1_ + *a*_1*i*_ denote the random intercept and slope of MD for i^th^ participant, let Y_1i_(t) and Y_2i_(t) denote MD and VA for i^th^ participant at time t, then the joint distribution of {Y_1i_(t), Y_2i_(t), γ_0i_, γ_1i_} at given time t also follows a normal distribution,

$$ \left(\begin{array}{c}\hfill {\mathrm{Y}}_{1\mathrm{i}}(t)\hfill \\ {}\hfill {\mathrm{Y}}_{2\mathrm{i}}(t)\hfill \\ {}\hfill {\gamma}_{0\mathrm{i}}\hfill \\ {}\hfill {\gamma}_{1\mathrm{i}}\hfill \end{array}\right)\sim N\left(\left(\begin{array}{c}\hfill {Z}_i{\alpha}_Z+{\alpha}_0+{\alpha}_1t\hfill \\ {}\hfill {Z}_i{\beta}_Z+{\beta}_0+{\beta}_1t\hfill \\ {}\hfill {Z}_i{\alpha}_Z+{\alpha}_0\hfill \\ {}\hfill {\alpha}_1\hfill \end{array}\right),\kern0.5em \left(\begin{array}{cccc}\hfill {\nu}_{11}(t)\hfill & \hfill {\nu}_{12}(t)\hfill & \hfill {\nu}_{13}(t)\hfill & \hfill {\nu}_{14}(t)\hfill \\ {}\hfill \hfill & \hfill {\nu}_{22}(t)\hfill & \hfill {\nu}_{23}(t)\hfill & \hfill {\nu}_{24}(t)\hfill \\ {}\hfill \hfill & \hfill \hfill & \hfill {\nu}_{33}\hfill & \hfill {\nu}_{34}\hfill \\ {}\hfill \hfill & \hfill \hfill & \hfill \hfill & \hfill {\nu}_{44}\hfill \end{array}\right)\right) $$, with

$$ {\displaystyle \begin{array}{l}{\upsilon}_{11}\left(\mathrm{t}\right)={\sigma}_{a_0}^2+{t}^2{\sigma}_{a_1}^2+2t{\sigma}_{a_0{a}_1}+{\sigma}_1^2,\kern0.5em \\ {}{\upsilon}_{22}\left(\mathrm{t}\right)={\sigma}_{b_0}^2+{t}^2{\sigma}_{b_1}^2+2t{\sigma}_{b_0{b}_1}+{\sigma}_2^2,\kern0.75em \\ {}{\upsilon}_{12}\left(\mathrm{t}\right)={\sigma}_{a_0{b}_0}+t{\sigma}_{a_0{b}_1}+t{\sigma}_{a_1{b}_0}+{t}^2{\sigma}_{a_1{b}_1},\kern0.75em \\ {}{\upsilon}_{13}\left(\mathrm{t}\right)={\sigma}_{a_0}^2+t{\sigma}_{a_0{a}_1},\kern2em {\upsilon}_{14}\left(\mathrm{t}\right)={\sigma}_{a_0{a}_1}+t{\sigma}_{a_1}^2,\\ {}{\upsilon}_{23}\left(\mathrm{t}\right)={\sigma}_{a_0{b}_0}+t{\sigma}_{a_0{b}_1},\kern1.5em {\upsilon}_{24}\left(\mathrm{t}\right)={\sigma}_{a_1{b}_0}+t{\sigma}_{a_1{b}_1},\\ {}{\upsilon}_{33}={\sigma}_{a_0}^2,\kern1em {\upsilon}_{34}={\sigma}_{a_0{a}_1},\kern0.75em \mathrm{and}\kern0.5em {\upsilon}_{44}={\sigma}_{a_1}^2.\end{array}} $$

Once the bivariate linear mixed model has been fitted and its variance-covariance matrix is obtained, the joint distribution of {Y_1i_(t), Y_2i_(t), γ_0i_, γ_1i_} at given time t can be obtained by plugging in the estimated parameters. The conditional correlation at a given time t is denoted as ρ(Y_1i_(t), Y_2i_(t)| γ_0i_ > c_1_, γ_1i_ > c_2_), where c_1_ and c_2_ represent clinical relevant thresholds for the intercept and slope of MD respectively. In the clinical practice of POAG management, an MD level of -5 dB or above marks a mild damage and MD < −5 dB is deemed as moderate/advanced damage. An MD slope of −1 dB/year is often regarded as clinically significant deterioration. Since this paper only includes participants with newly diagnosed POAG, it is believed that a change of −0.5 dB/year is also worthy close attention [15]. We therefore choose c_1_ = −5 dB and c_2_ = −0.5 dB/years throughout the remainder of this paper. Once the parameters from bivariate mixed models have been determined, the conditional correlation ρ(Y_1i_(t), Y_2i_(t)| γ_0i_ > c_1_, γ_1i_ > c_2_) can be estimated using parametric bootstrap resampling method with the following 4 steps.

1. At a given time t, a random sample of *N* = 269 subjects is generated from the above multivariate normal distributions, N(μ_i_(t), ∑(t)), with *i* = 1, 2, …, 269. Each dataset includes 4 variables, MD (Y_1i_), VA (Y_2i_), intercept of MD (γ_0i_), and slope of MD (γ_1i_).
2. Pearson’s correlation coefficient between MD and VA is calculated among these subjects who satisfy the conditions (i.e., with MD at diagnosis > −5 dB and slope of MD > −0.5 dB/year).
3. The above 2 steps are repeated 10,000 times to obtain the average conditional correlation at time t. The 95% confidence interval is also estimated as the 2.5 and 97.5 percentiles of the resultant correlations.
4. The above steps are repeated over different time points to describe the change of marginal correlations over time.

The assumption of independent errors in the above model could be relaxed if necessary [6]. In the case of correlated errors, $$ \left(\begin{array}{c}\hfill {\varepsilon}_{1i}\hfill \\ {}\hfill {\varepsilon}_{2i}\hfill \end{array}\right)\sim N\left(\left(\begin{array}{c}\hfill 0\hfill \\ {}\hfill 0\hfill \end{array}\right),\left(\begin{array}{cc}\hfill {\sigma}_1^2\hfill & \hfill {\sigma}_{12}\hfill \\ {}\hfill {\sigma}_{12}\hfill & \hfill {\sigma}_2^2\hfill \end{array}\right)\right) $$, the formula for correlations between random effects remain unchanged, but the marginal correlation over time is calculated as follows and the term *v*_12_(*t*) for conditional marginal correlation also needs to be updated accordingly,

$$ {\displaystyle \begin{array}{l}{\rho}_{\mathrm{marginal}}(t)=\frac{\sigma_{a_0{b}_0}+t{\sigma}_{a_0{b}_1}+t{\sigma}_{a_1{b}_0}+{t}^2{\sigma}_{a_1{b}_1}+{\sigma}_{12}}{\sqrt{\sigma_{a_0}^2+2t{\sigma}_{a_0{a}_1}+{t}^2{\sigma}_{a_1}^2+{\sigma}_1^2}\sqrt{\sigma_{b_0}^2+2t{\sigma}_{b_0{b}_1}+{t}^2{\sigma}_{b_1}^2+{\sigma}_2^2}},\\ {}\ \mathrm{and}\kern0.5em {\upsilon}_{12}\left(\mathrm{t}\right)={\sigma}_{a_0{b}_0}+t{\sigma}_{a_0{b}_2}+t{\sigma}_{a_2{b}_0}+{t}^2{\sigma}_{a_2{b}_2}+{\sigma}_{12}.\end{array}} $$

In this article, the 95% confidence interval (CI) of conditional marginal correlation was estimated using 10,000 parametric bootstrap samples, while the 95% CIs of other correlations were obtained from 500 empirical bootstrap samples using participants as resampling units. All the BLMMs were fitted using the MIXED procedure in SAS statistical package 9.4 (SAS Institutes, Cary, NC).

## Results

### Application to ocular hypertension treatment study (OHTS)

Table 1 showed the summary statistics for baseline demographic characteristics, the estimated regression coefficients, and the estimated parameters of the variance-covariance from univariate mixed model to each outcome. Among these baseline covariates, age was significantly associated with both outcomes and these elderly participants had poor outcomes. Those participants with higher intraocular pressure (IOP) at diagnosis or with lower central corneal thickness (CCT) had significantly worse MD. Participants self-identified as African-American also tended to be worse in both outcomes though the difference was only significant in VA. Note that all the continuous variables (Age, CCT, IOP, and HCD) were standardized to have mean 0 and variance 1 in all the models throughout this paper and hence the regression coefficients actually represented the effect per 1-SD change.

**Table 1:** Summary statistics of baseline covariates, the estimated regression coefficients, and the estimated parameters of variance (Var) and covariance (Cov) from the univariate and bivariate mixed models for longitudinal mean deviation (MD) and visual acuity (VA)

Variables	Mean ± SD or N (%)	Estimated fixed and random effects ± standard errors			
		Univariate mixed model		Bivariate mixed model	
		MD	VA	MD	VA
Fixed effects:					
Intercept	-	−2.26 ± 0.34^#^	50.75 ± 1.03^#^	−2.23 ± 0.34^#^	50.88 ± 1.00^#^
Slope	-	−0.35 ± 0.04^#^	−0.60 ± 0.11^#^	−0.35 ± 0.04^#^	−0.69 ± 0.11^#^
Age (years)	65.7 ± 9.5	−0.49 ± 0.15^#^	−3.45 ± 0.43^#^	−0.43 ± 0.14^#^	−3.07 ± 0.42^#^
IOP (mmHg)	22.3 ± 6.2	−0.55 ± 0.16^#^	−0.55 ± 0.47	−0.55 ± 0.16^#^	−0.40 ± 0.45
CCT (μm)	558.4 ± 37.8	0.35 ± 0.15^*^	−0.07 ± 0.45	0.35 ± 0.15^*^	−0.22 ± 0.44
HCD	0.53 ± 0.19	0.20 ± 0.15	0.12 ± 0.44	0.20 ± 0.15	0.12 ± 0.44
Male	150 (56%)	0.11 ± 0.29	0.77 ± 0.87	0.06 ± 0.29	1.01 ± 0.84
African American	89 (33%)	−0.57 ± 0.31	−2.13 ± 0.94^*^	−0.58 ± 0.31	−2.15 ± 0.91^*^
Observation group	158 (59%)	−0.08 ± 0.32	−0.26 ± 0.94	−0.10 ± 0.32	−0.69 ± 0.92
Random effects:^&^					
Var(E_k_)		2.03 ± 0.06^#^	19.15 ± 0.96^#^	2.02 ± 0.06^#^	19.19 ± 0.96^#^
Var(I_k_)		5.05 ± 0.51^#^	39.25 ± 4.69^#^	5.09 ± 0.51^#^	38.77 ± 4.63^#^
Var(S_k_)		0.29 ± 0.04^#^	1.35 ± 0.25^#^	0.29 ± 0.04^#^	1.35 ± 0.24^#^
Cov(I_k_, S_k_)		0.62 ± 0.10^#^	−1.23 ± 0.83	0.63 ± 0.10^#^	−0.73 ± 0.79
Cov(I_1_, I_2_)		-	-	-	3.88 ± 1.17^#^
Cov(S_1_, S_2_)		-	-	-	0.37 ± 0.08^#^
Cov(I_1_, S_2_)		-	-	-	1.16 ± 0.27^#^
Cov(I_2_, S_1_)		-	-	-	0.67 ± 0.31^*^

*IOP* intraocular pressure, *CCT* central corneal thickness, *HCD* horizontal cup-to-disc ratio

^&^E_k_, I_k_, S_k_: error term, random intercept, and random slope for MD (k = 1) and VA (k = 2)

^*^ *p* < 0.05, ^#^ *p* < 0.01

#### Primary analysis for the correlation between longitudinal MD and VA

Two bivariate linear mixed models (BLMM) were fitted to describe the relationship between MD and VA, one assuming correlated error terms and the other with independent errors. The likelihood ratio test showed that the model with independent errors would provide an adequate fit (X^2^ = 2.9, df = 1, *p* = 0.09). Hence, the BLMM with independent error terms was selected as the final model. The estimated fixed and random effects from this BLMM were presented in Table 1. The results showed that the univariate and bivariate mixed models produced very close estimates, especially the fixed effects. Following Fieuws et al. [6], the correlation between longitudinal MD and VA was summarized in terms of the association between subject-specific evolutions (as measured by random intercepts and slopes) as well as the evolution of association (as measured by marginal correlation over time). Table 2 presented the estimated correlation coefficients for random intercepts and slopes and their 95% confidence intervals (CI). There was a significant positive correlation in both random intercepts (ρ = 0.278, 95% CI: 0.121–0.420) and random slopes (ρ = 0.579, 95% CI: 0.349–0.810).

**Table 2:** Estimated correlation coefficients and their 95% confidence intervals in the random intercepts and random slopes, from the primary analysis and three sensitivity analyses using bivariate linear mixed models

Models	Correlations between random intercepts	Correlations between random slopes
Primary analyses: without adjusting heterogeneity	0.278 (0.121, 0.420)	0.579 (0.349, 0.810)
Sensitivity #1: adjusting the severity of vision damage at diagnosis (MD < −5 dB vs. MD ≥ −5 dB)	0.267 (0.090, 0.452)	0.519 (0.285, 0.753)
Sensitivity #2: excluding those who labelled as “Rapid Progression”.	0.217 (0.077, 0.354)	0.289 (0.010, 0.593)
Sensitivity #3: estimating class-specific intercepts and slopes	0.145 (−0.037, 0.324)	0.106 (−0.255, 0.490)

**Fig. 2:**
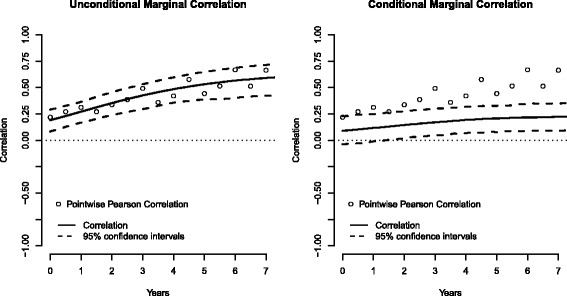
Estimated unconditional marginal correlation and conditional marginal correlation (given MD at diagnosis > −5 dB and slope of MD > −0.5 dB/year) in the OHTS data, where *solid lines* and *broken lines* represented the estimated correlation and its 95% confidence intervals respectively. Pointwise Pearson correlation at each time was also presented

Figure 2 plotted the marginal correlations (solid lines) and the corresponding 95% confidence intervals (broken lines). Pearson correlation coefficient estimated at each time point was also used as a naïve approximation to the marginal correlation over time. In general, the marginal correlation at diagnosis (time 0) approximated the correlation between random intercepts, and the marginal correlation converged to the correlation between random slopes as the time departures from 0 [6]. As comparing to the unconditional correlation, the conditional correlation in participants with stable vision function (i.e., given MD at diagnosis above −5 dB and MD slope > −0.5 dB/year) reduced substantially, indicating that the observed correlation between MD and VA was mainly induced by these participants with more deteriorating conditions. Figure 2 also revealed a considerable variation from time to time in the pointwise Pearson correlation, even after we had imposed the restriction so that the analysis cohort contained at least 30 participants at any given time point.

#### Sensitivity analyses

Several sensitivity analyses were further performed to better understand the impact of heterogeneity on assessing correlations. The disease heterogeneity was approximated by 4 subpopulations from a previous study using the same OHTS dataset [16]. Briefly, the trajectories of MD and pattern standard deviation (PSD, another global index of visual field tests) were summarized by a non-parametric functional principal component (FPC) analysis [17], and a latent class analysis (LCA) was performed to the first FPC scores to identify subgroups (latent classes) of individuals with distinct patterns of MD and PSD trajectories. Unlike a linear mixed model that described the individual trajectories by assuming them as random effects following a continuous distribution, the LCA approach used latent classes (unobserved trajectory groups) as an approximation of the unknown distribution. In the other words, the latent classes could be thought of as longitudinal strata where population variability was captured by differences across groups in the shape and level of their trajectories [13].

Table 4 summarized the class-specific intercepts and slopes for MD and VA respectively, after adjusting the baseline covariates. It showed that the profiles in the 4^th^-class were substantially different from the other classes. Since MD < −5 dB marks a moderate/advanced vision damage and a deteriorating rate of −0.5 dB/year is deemed clinically significant in newly diagnosed POAG [15], we therefore labeled Classes 1 to 4 as “Stable, High MD”, “Stable, Low MD”, “Progression”, and “Rapid Progression”, respectively. Following 3 BLMMs were fitted as sensitivity analyses. The average correlation coefficients between random effects and their 95% confidence intervals from each model were presented in Table 2. The estimated parameters of variance and covariance from each model were also presented in Table 3.

- ***Sensitivity Analysis #1:*** *adjusting the severity of vision damage at diagnosis*. The first BLMM incorporated an indicator for moderate/advanced glaucoma (MD < −5 dB) into the primary analysis. The model lead to similar estimates as these from primary analysis, though the strength of estimated correlation in the sensitivity analysis decreased slightly towards null.
- ***Sensitivity Analysis #2:*** *excluding those participants who are labeled as “Rapid Progression”*. The 2^nd^ BLMM was fitted after excluding these 16 participants who were labeled as “Rapid Progression”. Although these participants only accounted for 6% of total sample size, excluding them lead to a substantial decrease in the estimated correlation, especially between random slopes.
- ***Sensitivity Analysis #3:*** *full adjustment of heterogeneity.* The 3^rd^ sensitivity analysis fit a BLMM including both class-specific intercepts and class-specific slopes. The results showed that none of the correlation was significantly different from 0, indicating a conditional independence between MD and VA given the heterogeneity of MD trajectory.

**Table 3:** Estimated parameters of variance (Var) and covariance (Cov) from the primary analysis and the three sensitivity analyses using bivariate linear mixed models

Parameters for variance-covariance^&^	Primary analysis	Sensitivity analysis #1	Sensitivity analysis #2	Sensitivity analysis #3
Var(E_1_)	2.02 ± 0.06^#^	1.99 ± 0.06^#^	1.44 ± 0.05^#^	2.03 ± 0.06^#^
Var(E_2_)	19.19 ± 0.96^#^	19.21 ± 0.96^#^	17.03 ± 0.86^#^	19.30 ± 0.96^#^
Var(I_1_)	5.09 ± 0.51^#^	2.98 ± 0.34^#^	2.69 ± 0.28^#^	1.65 ± 0.20^#^
Var(I_2_)	38.77 ± 4.63^#^	39.26 ± 4.78^#^	36.12 ± 4.33^#^	36.50 ± 4.41^#^
Var(S_1_)	0.29 ± 0.04^#^	0.19 ± 0.03^#^	0.12 ± 0.02^#^	0.09 ± 0.02^#^
Var(S_2_)	1.35 ± 0.24^#^	1.31 ± 0.24^#^	0.73 ± 0.16^#^	0.81 ± 0.18^#^
Cov(I_1_, S_1_)	0.63 ± 0.10^#^	0.14 ± 0.07^*^	0.02 ± 0.05	−0.11 ± 0.04^#^
Cov(I_2_, S_2_)	−0.73 ± 0.79	−1.11 ± 0.81	−1.11 ± 0.63	−1.13 ± 0.69
Cov(I_1_, I_2_)	3.88 ± 1.17^#^	3.01 ± 0.98^#^	2.14 ± 0.79^#^	1.13 ± 0.69
Cov(S_1_, S_2_)	0.37 ± 0.08^#^	0.26 ± 0.06^#^	0.09 ± 0.04^*^	0.03 ± 0.04
Cov(I_1_, S_2_)	1.16 ± 0.27^#^	0.53 ± 0.21^*^	0.13 ± 0.15	0.13 ± 0.14
Cov(I_2_, S_1_)	0.67 ± 0.31^*^	0.44 ± 0.27	0.28 ± 0.19	0.40 ± 0.19^*^

^&^E_k_, I_k_, S_k_: error term, random intercept, and random slope for MD (k = 1) and VA (k = 2)

^*^ *p* < 0.05, ^#^ *p* < 0.01

In summary, the results revealed that the assumption of homogeneous population in BLMM is important for assessing correlation between multivariate longitudinal data. As illustrated in different sensitivity analyses, the estimated correlations could be substantially distorted in the presence of unobserved heterogeneity. In general, the adjustment of heterogeneity tended to reduce the strength of correlations, but all the estimates had a relatively wide range of 95% confidence interval, especially between random slopes. In such a real-world data analysis, however, we do not know the “true” population parameters and the estimates could be influenced by sampling errors. In the section follows, simulated data were used to quantify to what extent the “true” correlation between longitudinal outcomes could be distorted due to heterogeneity.

### Simulation study

The simulated data were a mixture of stable and progressive subjects, generated from following bivariate linear mixed models,

$$ \left(\begin{array}{c}\hfill {\mathrm{Y}}_{1\mathrm{i}}^{\mathrm{g}}(t)\hfill \\ {}\hfill {\mathrm{Y}}_{2\mathrm{i}}^{\mathrm{g}}(t)\hfill \end{array}\right)\kern0.5em =\left(\begin{array}{c}\hfill {\alpha}_0^{\mathrm{g}}+{\alpha}_1^{\mathrm{g}}t\hfill \\ {}\hfill {\beta}_0^{\mathrm{g}}+{\beta}_1^{\mathrm{g}}t\hfill \end{array}\right)+\left(\begin{array}{c}\hfill {a}_0+{a}_1t\hfill \\ {}\hfill {b}_0+{b}_1t\hfill \end{array}\right)+\left(\begin{array}{c}\hfill {\varepsilon}_{1i}\left(\mathrm{t}\right)\hfill \\ {}\hfill {\varepsilon}_{2i}(t)\hfill \end{array}\right), $$

$$ \mathrm{with}\kern1em \left[\begin{array}{c}\hfill {a}_{0i}\hfill \\ {}\hfill {a}_{1i}\hfill \\ {}\hfill {b}_{0i}\hfill \\ {}\hfill {b}_{1i}\hfill \end{array}\right]\kern0.5em \sim N\left(\ \left[\begin{array}{c}\hfill 0\hfill \\ {}\hfill 0\hfill \\ {}\hfill 0\hfill \\ {}\hfill 0\hfill \end{array}\right],\varSigma =\left[\begin{array}{cccc}\hfill 1.65\hfill & \hfill 1.13\hfill & \hfill -0.11\hfill & \hfill 0.13\hfill \\ {}\hfill \hfill & \hfill 36.5\hfill & \hfill 0.4\hfill & \hfill -1.13\hfill \\ {}\hfill \hfill & \hfill \hfill & \hfill 0.09\hfill & \hfill 0.03\hfill \\ {}\hfill \hfill & \hfill \hfill & \hfill \hfill & \hfill 0.81\hfill \end{array}\right]\right), $$

$$ \mathrm{and}\kern0.5em \left[\begin{array}{c}\hfill {\varepsilon}_{1i}\hfill \\ {}\hfill {\varepsilon}_{2i}\hfill \end{array}\right]\sim N\left(\left[\begin{array}{c}\hfill 0\hfill \\ {}\hfill 0\hfill \end{array}\right],\kern0.5em {\varSigma}_{\mathrm{e}}=\left[\begin{array}{cc}\hfill 2.0\hfill & \hfill \rho \sqrt{2}\sqrt{19}\hfill \\ {}\hfill \rho \sqrt{2}\sqrt{19}\hfill & \hfill 19.0\hfill \end{array}\right]\right). $$

- The group indicator (g = 1, 2) represented stable and progressive participants, and the fixed-effect parameters $\left\{{\alpha}_0^g,{\alpha}_1^g,{\beta}_0^g,{\beta}_1^g\right\}$were retrieved from the estimated parameters in the 2nd- and 4th-class (Table 4), with $\left\{{\alpha}_0^1,{\alpha}_1^1,{\beta}_0^1,{\beta}_1^1\right\}=\left\{-2,53,\hbox{-} 0.2,\hbox{-} 0.4\right\}$ and $\left\{{\alpha}_0^2,{\alpha}_1^2,{\beta}_0^2,{\beta}_1^2\right\}=${‐8, 48, ‐2.0, ‐3.0} respectively.
- The parameters for random effects *Σ* and *Σ*_*e*_ were obtained from BLMM in the 3rd sensitivity analysis and assumed common across the two mixture groups.
- t = {0, 0.5, …, 5} denoted the semi-annual measuring times and we assumed that all individuals had exactly the same follow-up time in this simulation.
- Unless otherwise specified, we assumed independent error terms (i.e.,*ρ* = 0) in all the simulations.

**Table 4:** The trajectory profiles of longitudinal mean deviation (MD) and visual acuity (VA) across latent classes, after accounting for other baseline demographic and clinical characteristics

Parameters	N (%)	MD	VA
Intercept:			
Class1 (reference)	83 (31%)	0.08 ± 0.21	54.66 ± 0.97
Class2	117 (43%)	−1.73 ± 0.23^#^	52.94 ± 1.08
Class3	53 (20%)	−3.11 ± 0.28^#^	52.89 ± 1.33
Class4	16 (6%)	−8.42 ± 0.45^#^	48.15 ± 2.17^#^
Slope:			
Class1 (reference)	83 (31%)	−0.08 ± 0.05	−0.33 ± 0.17
Class2	117 (43%)	−0.15 ± 0.06	−0.35 ± 0.23
Class3	53 (20%)	−0.54 ± 0.07^#^	−0.86 ± 0.26^*^
Class4	16 (6%)	−2.04 ± 0.13^#^	−3.37 ± 0.47^#^

^*^ *p* < 0.05; ^#^ *p* < 0.01

Each simulation generated 500 random samples and each sample consisted 300 subjects. In each simulation, the average correlation coefficients were calculated for both marginal correlation (unconditional and conditional) and correlation between random effects. Three scenarios were considered to assess to what extent the true correlation could be distorted in the presence of heterogeneity or model misspecification.

**Fig. 3:**
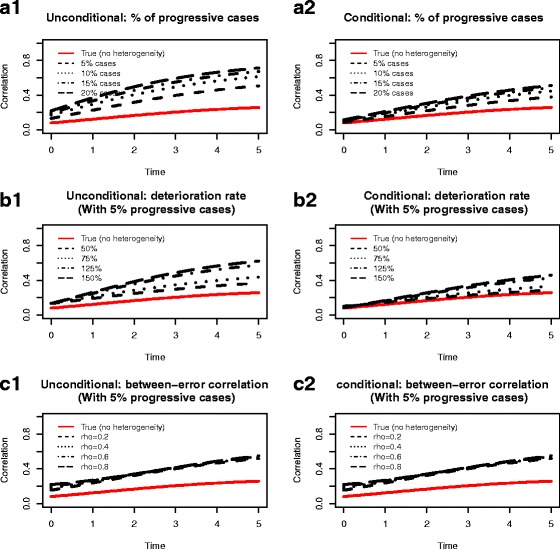
Estimated unconditional marginal correlation and conditional marginal correlation (given MD at diagnosis > −5 dB and slope of MD > −0.5 dB/year) under 3 simulated scenarios. Scenarios **a**: different proportions of progressive cases; Scenarios **b**: different magnitudes of deteriorating rates in progressive cases; Scenarios **c**: various strength of between-error correlations

1. **Scenarios A:** *Impact of size of heterogeneity*. In the simulated data, the progressive subjects (i.e., those with g = 2) accounted for 5%, 10%, 15% and 20% of total sample size respectively. Figure 3 A1 showed the estimated average marginal correlation over time under different proportions of progressive cases. We saw that even the presence of a small fraction of heterogeneity could dramatically distort the estimated correlation and, as expected, greater proportion of progressive cases imposed stranger influence. In contrast, the influence of heterogeneity on the conditional correlation was much smaller, and the impact was almost ignorable in the presence of only small fraction of progressive cases (Fig. 3 A2). The results also showed that the presence of heterogeneity could lead to substantial overestimation of correlations between random effects, especially in the random slopes (Table 5).
2. **Scenarios B:** *Impact of magnitude of heterogeneity*. In each simulation, the progressive subjects accounted for only 5% of the total sample size, but the slopes in the progressive subjects varied as 50%, 75%, 125% and 150% of what were used under Scenario A. Figure 3 B1 showed that a higher magnitude of deterioration (like that of 4th-class in the OHTS data) could substantially distort the marginal correlations over time while a lower deterioration (like that of 3rd-class in the OHTS data) would have little impact on the estimated correlation. Again, the estimated conditional correlation was relatively robust to the presence of rapid progressive cases (Fig. 3 B2). The results also showed that higher deteriorating rate could lead to substantial overestimation of correlations between random slopes (Table 5).
3. **Scenarios C:** *Impact of correlation between error terms*. Since a candidate BLMM in the primary analysis showed a weak correlation between error terms (ρ = 0.07) with a trend towards significance (*p* = 0.09), we also assessed the potential impact of independence assumption of error terms on correlation. In each simulated dataset, the progressive subjects accounted for only 5% of the total sample size and the slopes of progressive subjects were the same as those under Scenario A. The data were generated under a variety degree of correlations (*ρ* = 0.2, 0.4, 0.6, 0.8) between error terms, but analyzed assuming independent errors. The result showed that both the unconditional and conditional marginal correlations were rather insensitive to the degree of correlation between error terms (Fig. 3 C1 and C2). However, ignoring the correlation between error terms could lead to a substantial overestimation of correlations between random effects, especially in the random slopes. The impact was trivial when there was only a weak correlation between error terms, but became much strong in the presence of moderate/high between-error correlations (Table 5).

**Table 5:** Averages and 95% confidence intervals for correlations between random intercepts and correlation between random slopes based on simulated data, where data were generated under 3 different scenarios (Scenarios A: different proportions of progressive cases; Scenarios B: different magnitudes of deteriorating rates in progressive cases; Scenarios C: various strength of between-error correlations)

Simulation Scenarios	Correlation between random intercepts	Correlation between random slopes
True correlation (no heterogeneity)	0.145	0.111
Scenario A:		
5%	0.199 (0.070, 0.329)	0.522 (0.390, 0.654)^a^
10%	0.246 (0.118, 0.373)	0.657 (0.562, 0.753)^a^
15%	0.268 (0.153, 0.383)^a^	0.727 (0.651, 0.804)^a^
20%	0.296 (0.178, 0.413)^a^	0.769 (0.698, 0.840)^a^
Scenario B:		
50%	0.200 (0.070, 0.329)	0.240 (0.051, 0.429)
75%	0.207 (0.077, 0.336)	0.382 (0.223, 0.542)^a^
100%	0.199 (0.070, 0.329)	0.522 (0.390, 0.654)^a^
125%	0.205 (0.078, 0.332)	0.634 (0.532, 0.734)^a^
150%	0.202 (0.078, 0.327)	0.706 (0.616, 0.795)^a^
Scenario C:		
0.0	0.199 (0.070, 0.329)	0.522 (0.390, 0.654)^a^
0.2	0.232 (0.104, 0.361)	0.603 (0.479, 0.727)^a^
0.4	0.273 (0.147, 0.398)^a^	0.686 (0.574, 0.797)^a^
0.6	0.303 (0.180, 0.425)^a^	0.769 (0.664, 0.873)^a^
0.8	0.334 (0.218, 0.450)^a^	0.853 (0.756, 0.949)^a^

^a^95% confidence interval does not contain the true value

In summary, the simulated data confirmed the relative robustness of conditional correlation to potential heterogeneity and indicated that these participants in the Class 4 were more likely strong influential cases for assessing the correlation between longitudinal MD and VA. The sensitivity analysis excluding these 16 participants (who only account for 6% of the total samples) resulted in substantial decrease of correlation.

## Discussion

In clinical trials and epidemiologic studies, it is quite common to have two or more outcomes measured repeatedly over time. These multivariate outcomes are likely to be correlated and the statistical analysis often requires taking such associations into account. A number of approaches for analyzing multivariate longitudinal data have been proposed in the statistical literature, ranging from the most naïve approach of ignoring the association to the full joint model that specifies the joint distribution and correlation structure among different outcomes [1]. The choice for a specific type of model is often guided by the specific characteristics of data such as the structure of data (balance or unbalanced), the scale of outcome measures (continuous, ordinal, or binary), as well as the research questions of interest (the average evolution over time or the association structure) [11]. When the average evolution over time (fixed effects) is of primary interest, for example, specification of the full joint distribution may be avoided by using a generalized estimating equation (GEE) approach where association structure is treated as a nuisance, and a valid inference regarding fixed effects can still be obtained even when the within-subject associations are mis-specified. When the primary interests focus on the association structure itself, a full joint model of outcomes is often preferred [2]. Among various joint models, multivariate random-effect mixed models provide a versatile tool in estimating the association among different outcomes as well as how the association evolves over time. These models do not require a balanced data structure and thus allow each subject to have different number of observations taken at different time points. They also enable reducing the effect of measurement error attenuation in estimating the correlation between the rates of change over time in different growth curves [1]. However, as illustrated by the OHTS data and simulated data, the assumption of homogeneous population in such a multivariate random-effect mixed model is important for estimation and inference about correlation. The estimated correlation could be substantially distorted even in the presence of a small proportion of heterogeneity.

In this paper, we demonstrated various strategies to relax the rather restrictive one-size-fit-all assumption in the bivariate linear mixed models. We showed that conditional correlation given random effects provides a robust estimate to describe the correlation over time in the presence of unobserved heterogeneity. The conditional correlation works better when there is only small fraction of strong influential cases and/or when the difference in trajectories is not so massive among heterogeneous cases. The study also revealed that latent class analysis approach provides a useful tool to explore disease heterogeneity and to assess whether the individual trajectories are smoothly distributed around the population average. Since the existence of latent classes can be because of real heterogeneity (mixture of distinct subpopulations) or simply due to non-normality distribution [13], we therefore expect that the incorporation of latent classes into mixed model could also improve normality assumption. In the presence of massive heterogeneity, however, the assumption of multivariate normality distribution in BLMM is violated and all the estimated correlations including conditional ones will be substantially distorted. In such a case, the dual-trajectory model [18] may provide a better alternative to describe the connections between developmental trajectories of two longitudinal outcomes. It allows the trajectories to evolve contemporaneously or over different non-overlapping time periods. The correlation between different outcomes is represented by the conditional probabilities (i.e., the chance of a given trajectory in one outcome conditional on the trajectory of another) or the joint probabilities (i.e., the chance for a given co-trajectory).

Substantively, our study showed that there is a significant positive correlation between longitudinal MD and VA, and that the correlation constantly strengthens as the time increased. However, further analysis revealed that the correlation is induced primarily by participants with rapid deteriorating MD who only accounted for a small fraction of the total sample size. This finding was consistent to the recent observation that the overall average of VA in participants with newly diagnosis POAG was not significantly different from those without POAG [19].

Our study had some limitations. In this study we only assessed the potential impact of heterogeneity. As long as the estimation and inference on correlation are of primary interest, the results can also be influenced by a variety of other assumptions such as linearity and homoscedasticity. For this consideration, our analysis cohort was only restricted to the post-diagnosis data to better fulfil the linearity assumption, and the baseline demographic and clinical characteristics were included in the mixed models to reduce the unexplained variance. Another potential issue is the influence of missing data. Although these joint mixed models do not require a balanced data structure, they assume that data are missing at random. Inclusion of cases with greater attrition rates may weaken statistical precision and potentially introduce bias if such an assumption is incorrect. To reduce the potential influence of cases with greater attrition rates, in this study we only included data from these visits with at least 30 subjects.

## Conclusion

Bivariate linear mixed model (BLMM) is a versatile tool with regard to assessing correlation between multivariate longitudinal data and the conditional correlation given random effects provides a robust estimate to describe the correlation in the presence of unobserved heterogeneity.

## Abbreviations

BLMM: bivariate linear mixed model
CCT: central corneal thickness;
FPC: functional principal component
GEE: generalized estimating equation;
HCD: horizontal cup/disc ratio
IOP: intraocular pressure;
LCA: latent class analysis
MD: mean deviation;
OHTS: Ocular Hypertension Treatment Study
POAG: primary open angle glaucoma
VA: visual acuity
